# Effect of Bi^3+^ ion on upconversion-based induced optical heating and temperature sensing characteristics in the Er^3+^/Yb^3+^ co-doped La_2_O_3_ nano-phosphor

**DOI:** 10.1039/c8ra07438k

**Published:** 2018-10-09

**Authors:** R. S. Yadav, Dinesh Kumar, A. K. Singh, Ekta Rai, S. B. Rai

**Affiliations:** Department of Physics, Institute of Science, Banaras Hindu University Varanasi 221005 India sbrai49@yahoo.co.in; School of Materials Science and Technology, Indian Institute of Technology (Banaras Hindu University) Varanasi 221005 India akhilesh_bhu@yahoo.com

## Abstract

The upconversion-based optical heating and temperature sensing characteristics are investigated in the Er^3+^/Yb^3+^/Bi^3+^ tri-doped La_2_O_3_ nano-phosphor synthesized through a solution combustion method. The structural measurements reveal an increase in lattice parameters and particles size of the phosphor on increasing the concentrations of Bi^3+^ ions. The energy dispersive spectroscopic (EDS) measurements confirm the presence of La, Er, Yb, Bi and O elements in the tri-doped phosphor. The absorption spectra show the large number of bands due to Er^3+^, Yb^3+^ and Bi^3+^ ions. The Er^3+^/Yb^3+^ co-doped phosphor gives strong green emission bands at 523 and 548 nm upon 976 nm excitation due to ^2^H_11/2_ → ^4^I_15/2_ and ^4^S_3/2_ → ^4^I_15/2_ transitions of Er^3+^ ion, respectively. The emission intensity of these bands is enhanced upto 15 times in the presence of Bi^3+^ ions. The emission intensities of the 523 and 548 nm bands vary non-linearly with the pump power. The fluorescence intensity ratio (FIR) of the thermally coupled 523 and 548 nm emission bands shows efficient optical heating in the tri-doped phosphor. The FIR of the 523 and 548 nm emission bands further varies with the increase in temperature of the phosphor. The relative temperature sensing sensitivity has been calculated to be 71 × 10^−4^ K^−1^ at 450 K for the tri-doped phosphor. Thus, the Er^3+^/Yb^3+^/Bi^3+^ tri-doped La_2_O_3_ nano-phosphor may provide a platform to use it in the photonic devices, as an optical heater and temperature sensor.

## Abstract 2

The Er^3+^/Yb^3+^/Bi^3+^ tri-doped La_2_O_3_ nano-phosphor gives efficient induced optical heating and temperature sensing. This study is useful to understand these characteristics in different materials with various pump powers and temperatures.

## Graphical abstract

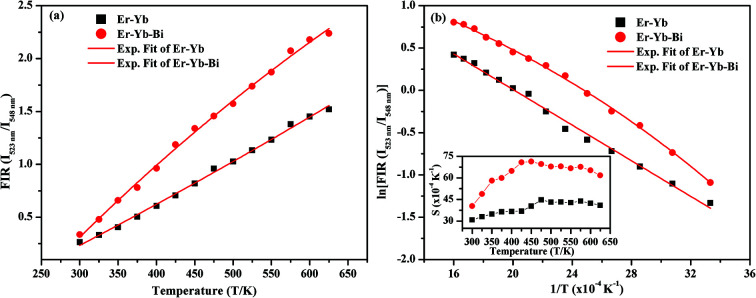

## 1. Introduction

Upconversion-based lanthanide spectroscopic studies have become a fascinating area of research in recent years due to its wide applications in various related fields, such as optical devices, optical heaters, photo-thermal treatment, temperature sensors, bio-imaging, *etc.*^1–9^ The thermally coupled energy levels in some of the lanthanide ions are of great scientific importance as they can sense a small change in the temperature around them even at a distance without any contact.^4–6,10^ These thermally coupled energy levels produce optical heating as well as being used for temperature sensing in the lanthanide doped phosphor materials. Er^3+^ is one such lanthanide ion, which contains thermally coupled energy levels *viz.*^2^H_11/2_ and ^4^S_3/2_ levels, separated by an energy gap of 800 cm^−1^. This energy gap can be covered by the phonon energy of the host lattice.^4–6,11^ The population of the ions in these two levels strongly depends on the phonon energy of the host. Thus, this causes a variation in intensity of the emitted bands from the thermally coupled levels in the Er^3+^/Yb^3+^ co-doped phosphor materials with increase in the pump power and temperature, thereby gives a dual characteristic such as optical heating and temperature sensing.

The optical properties of the Er^3+^/Yb^3+^ co-doped phosphor materials have been studied in detail by different groups.^12–20^ They have reported intense green and weak red upconverted emissions upon 976 nm excitation in which the green emission arises due to the transition from the thermally coupled energy levels (*viz.*^2^H_11/2_ and ^4^S_3/2_) to the ground level (^4^I_15/2_) of Er^3+^ ion. Since the two levels are thermally coupled, therefore, they can sense a variation as the input pump power of 976 nm source is changed. The emission intensity of one level increases whereas that of the other level decreases with the increase in the pump power. Therefore, the emission intensity ratio of the peaks arising from these levels, so called the fluorescence intensity ratio (FIR), is a measure of induced optical heating in the phosphor materials. The FIR of the peaks also varies with the increase in temperature of the sample. The optical heating and temperature sensing abilities of the Er^3+^/Yb^3+^ co-doped phosphors have also been investigated in different host materials.^4–6,11,19,21–23^ They have reported that the FIR is host dependent and the material with low phonon frequency can sustain better FIR with the pump power and temperature.

The research efforts are still continued to develop materials to improve the optical heating and temperature sensing abilities of the Er^3+^/Yb^3+^ co-doped phosphor materials. Some ions, such as Ho^3+^, Li^+^, Zn^2+^, Eu^3+^, *etc.* play a dynamic role for improving these characteristics when incorporated in the co-doped phosphors.^13,24–26^ These ions modify local crystal field around the lanthanide ions in such a way that they give relatively larger upconversion/fluorescence emission intensities on excitation with a near infrared (NIR) source. The effect of Li^+^ ion on optical heating and temperature sensing has been studied in the Er^3+^/Yb^3+^ co-doped Y_2_Ti_2_O_7_ phosphor and observed an enhancement in the emission intensity.^24^ The enhanced intensity from the Er^3+^ ion yields better FIR with the increase in the laser pump powers and temperatures. Mahata *et al.* have also reported the effect of Zn^2+^ in the Er^3+^/Yb^3+^ co-doped BaTiO_3_ phosphor and found improved properties of these characters.^25^ The emission intensity of the Er^3+^/Yb^3+^ co-doped Y_2_O_3_ phosphor is also affected by incorporating Eu^3+^ ion in it.^26^ The variation in the emission intensity alters the temperature sensing ability of the phosphor due to its energy transfer rate. Choudhary *et al.* have reported the effect of Bi^3+^ ion on the optical heating and temperature sensing in the Er^3+^/Yb^3+^ co-doped MgAl_2_O_4_ phosphor.^27^ However, the effect of Bi^3+^ ion on the optical heating and temperature sensing characteristics have not been studied in the Er^3+^/Yb^3+^ co-doped La_2_O_3_ phosphor to our knowledge.

In this paper, the La_2_O_3_ has been used as a host material due to its low phonon energy (∼400–650 cm^−1^). The low phonon energy host strongly supports the radiative transitions by reducing non-radiative relaxations.^8,11,24^ It is a very stable host, which can broaden the operating range and can be used for low and high temperature sensing. The Er^3+^ and Yb^3+^ have been used as lanthanide ions in which the Er^3+^ ion contains thermally coupled energy levels, which senses a slight change in the incident pump power and temperature. The Bi^3+^ ion has been incorporated in the co-doped phosphor to maintain strong crystal structure around the Er^3+^ ions. The Bi^3+^ is an interesting metal ion, which acts as sensitizer and activator and emits light ranging from UV to IR, even in mid-IR. The Bi based materials contain low phonon energy, which can reduce non-radiative transition rate significantly and favors upconversion.^28–30^ The increase in laser pump power leads to a difference in the emission intensities of the bands arising from the thermally coupled levels (*viz.*^2^H_11/2_ and ^4^S_3/2_). The FIR increases with the increase in the laser pump powers and also the sample temperatures. The Er^3+^/Yb^3+^/Bi^3+^ tri-doped La_2_O_3_ nano-phosphor is found to give very efficient induced optical heating and temperature sensing characteristics. This phosphor may open a new door for the study of optical heating and temperature sensing with a wide range of laser pump powers and temperatures.

## 2. Experimental

### 2.1 Materials and method

The Er^3+^/Yb^3+^/*x*Bi^3+^ tri-doped La_2_O_3_ nano-phosphor samples with *x* = 0, 5, 10, and 15 mol% were synthesized through a solution combustion method. The concentrations of Er^3+^ and Yb^3+^ ions are fixed at 0.7 and 3.0 mol%, respectively. The La_2_O_3_, Er_2_O_3_, Yb_2_O_3_ and Bi_2_O_3_ with 99.99% purity were used as starting materials. Urea has been used as a fuel for combustion. These materials were weighed in their stoichiometric ratios and dissolved in 5 ml of nitric acid. The solution thus obtained was diluted using de-ionized water under constant stirring followed by drop wise addition of urea. The final solution was stirred in a beaker at 60 °C until a homogeneous sticky gel was obtained and the water contents evaporate from the solution. The final product thus obtained was placed in a closed furnace maintained at a constant temperature (∼600 °C) for combustion. Various gases (CO_2_, N_2_, *etc.*) were released from the gel due to the exothermic reaction during combustion. As a result, the white fluffy powder was obtained. It was grinded further to get the fine powder. The phosphor was finally annealed at 1300 °C to improve the structural and optical properties of the samples. The annealed phosphors have been used for further measurements.

### 2.2 Characterization

The crystalline phase, crystallinity and crystal strain in the samples have been analyzed using X-ray diffraction (XRD) technique. The XRD patterns of the samples have been recorded using Cu, K_α_ radiation (*λ* = 0.15406 nm) from a MiniFlex 600 (Rigaku, Japan) unit at 2° min^−1^ scan speed. The Rietveld analysis of the XRD patterns of the samples was performed using FullProf Suite.^31^ The scanning electron microscope (SEM) has been used to record the morphological features of the samples with a Zeiss, Evo 18 Research unit. The elemental analysis of the samples was carried out using the energy dispersive spectroscopic (EDS) measurements. The Fourier transform infrared (FTIR) measurements of the phosphor samples were carried out using a Perkin Elmer IR spectrometer (FT-IR/FIR Frontier spectrometer) to verify different molecules present in the samples. The upconversion spectra of the phosphor samples have been monitored using 976 nm excitation from a diode laser and an iHR320, Horiba Jobin Yvon, monochromator attached with PMT (photomultiplier tube) at various input pump powers and temperatures.

## 3. Results and discussion

### 3.1 Structural analysis

#### 3.1.1 X-ray diffraction measurements

**Fig. 1:**
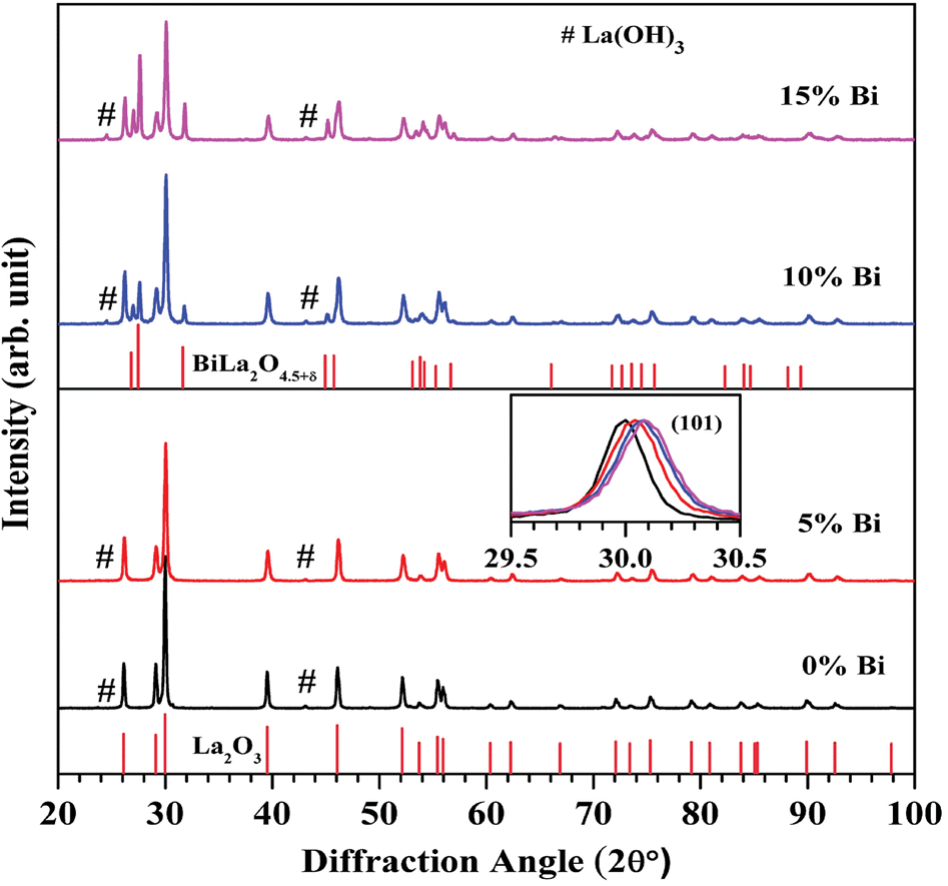
Room temperature XRD patterns of the Er^3+^/Yb^3+^/*x*Bi^3+^ tri-doped La_2_O_3_ nano-phosphor samples for different concentrations of Bi^3+^ ions (*i.e. x* = 0, 5, 10, and 15 mol%). The inset shows a shift in the XRD peak (101) towards higher angle side with increasing the concentrations of Bi^3+^ ions.

The room temperature X-ray diffraction (XRD) patterns of the Er^3+^/Yb^3+^/*x*Bi^3+^ tri-doped La_2_O_3_ nano-phosphor samples for different concentrations of Bi^3+^ ions (*i.e. x* = 0, 5, 10, and 15 mol%) were recorded in the 2*θ* range of 20–100° and they are shown in Fig. 1. It is found that the XRD patterns of the samples in absence and presence of 5 mol% concentrations of Bi^3+^ ions confirm the formation of a single phase of the hexagonal crystal structure of La_2_O_3_ with JCPDS File no. 05-0602 and space group *P*3̄*m*1. Since the La_2_O_3_ is hygroscopic in nature, therefore, it absorbs moisture present in the atmosphere.^8^ Due to this, a small amount of La(OH)_3_ phase with JCPDS file no. 13-0084 is also present in the XRD patterns (peaks marked by #). However, the phase of La_2_O_3_ is dominant over the La(OH)_3_ phase. When the concentrations of Bi^3+^ ions are increased to 10 and 15 mol% a small amount of an additional rhombohedral phase of BiLa_2_O_4.5+*δ*_ is also observed with JCPDS file no. 89-8058 and space group *R*3̄*m*. The inset in Fig. 1 indicates selected XRD peak (101) in the 2*θ* range of 29.5–30.5° in all the cases. The XRD peak (101) is shifted towards higher angle side with increasing concentrations of Bi^3+^ ions, which indicates a decrease in lattice parameters (lattice constants and unit cell volume). This was also confirmed by Rietveld refinement analysis of the phosphor samples.^31^

**Fig. 2:**
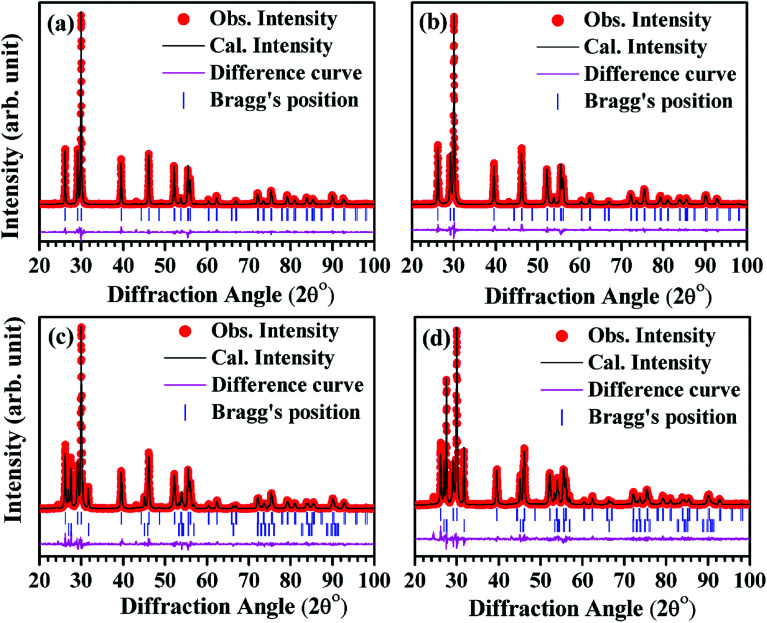
Rietveld refined patterns for the nano-phosphor samples with different concentrations of Bi^3+^ ions *i.e.* (a) 0 mol% (b) 5 mol% (c) 10 mol% and (d) 15 mol% at La^3+^ site.

For structural refinement, we selected pseudo-Voigt function to fit the XRD profiles, while the background of the patterns was modeled using linear interpolation method between the selected background points. In hexagonal phase of space group *P*3̄*m*1, we consider substitution of La^3+^/(0.7)Er^3+^/(3.0)Yb^3+^/(0–15)Bi^3+^ ions at site 2(d) (1/3, 2/3, *δz*), O^2−^(1) ions at site 2(d) (1/3, 2/3, 1/2 + *δz*) and O^2−^(2) ions at site 1(a) (0, 0, 0).^32^ During the structure refinement of second phase using rhombohedral crystal structure with *R*3̄*m* space group, we consider hexagonal setting in which the La^3+^/(10–15) Bi^3+^ ions occupy at site 3(a) (0, 0, 0), O^2−^(1) ions occupy at site (3a) (0, 0, *δz*) and O^2−^(2) ions occupy at site 3(a) (0, 0, 1/2 + *δz*).^33^Fig. 2(a–d) show Rietveld fits of the XRD patterns for different samples.

**Fig. 3:**
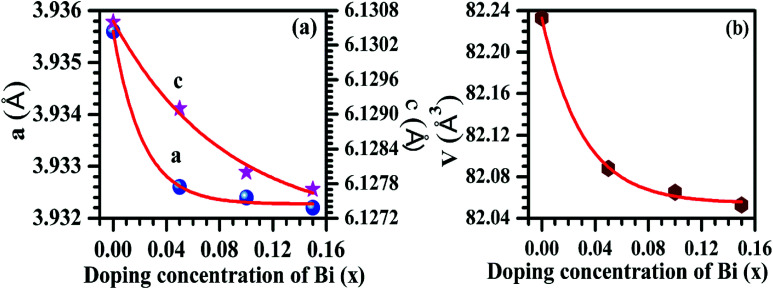
Variations of (a) lattice parameters and (b) unit cell volume as a function of different concentrations of Bi^3+^ ions (*i.e.* 0, 5, 10 and 15 mol%). The circular, stars and hexagon points show the refined values obtained after Rietveld refinement and the continuous lines indicate an exponentially fitted curve.

**Fig. 4:**
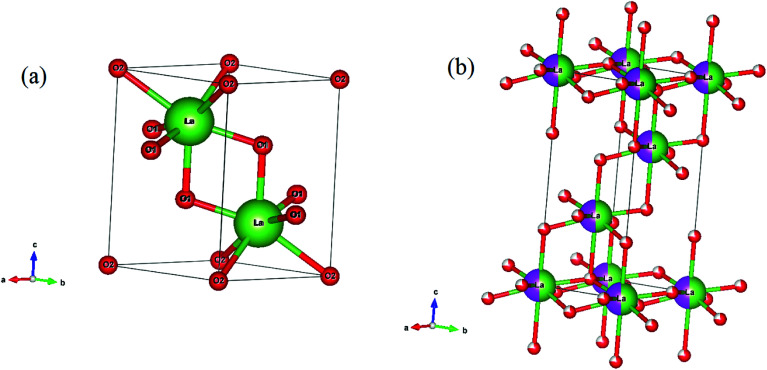
Ball and Stick model of the unit cells of space groups (a) *P*3̄*m*1 and (b) *R*3̄*m*.

**Fig. 5:**
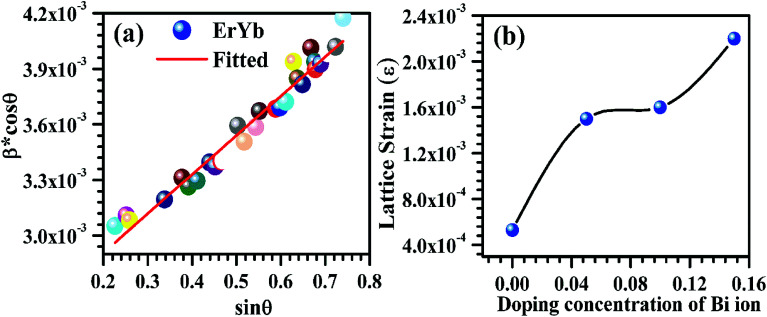
(a) A Williamson–Hall plot of the Er^3+^/Yb^3+^ co-doped La_2_O_3_ nano-phosphor sample and (b) a variation in lattice strain as a function of different concentrations of Bi^3+^ ions (*i.e.* 0, 5, 10 and 15 mol%).

In Rietveld fit, the circular solid points indicate an experimental data observed from the X-ray diffractometer, while the continuous solid line just overlapping the observed patterns shows calculated XRD profile. The lower continuous curve indicates difference between the observed and the calculated patterns, which clearly shows a very good fit between the patterns. The vertical bars represent the position of Bragg's reflections. The obtained Rietveld refined lattice parameters are summarized in Table 1. The lattice constant “*a*” decreases from *a* = *b* = 3.9356(1) Å to *a* = *b* = 3.9322(1) Å and the constant “*c*” also decreases from 6.1306(2) Å to 6.1277(3) Å, while the unit cell volume decreases from *V* = 82.233(2) Å^3^ to *V* = 82.053(6) Å^3^ in absence and presence of 15 mol% concentrations of Bi^3+^ ions for the space group *P*3̄*m*1, respectively. In the case of space group *R*3̄*m*, the refined lattice parameters also decrease with increasing concentrations of Bi^3+^ ions. The obtained refined lattice parameters were fitted using exponentially decaying function and found in a good agreement between the obtained and the fitted values. Fig. 3 reveals a variation of lattice parameters as a function of doping contents of Bi^3+^ ions for the space group *P*3̄*m*1. The lattice constant “*a*” varies according to the following expression:*a* = *a*_0_ + *a*_1_e^(−*x*/*t*)^where *a*_0_ and *a*_1_ are fitting constants with values 3.9323(1) Å and 0.003(2) Å, respectively; *t* is a decaying constant with its value 0.022(4) and *x* is the concentrations of Bi^3+^ ions. The lattice constant “*c*” decreases exponentially with the following equation:*c* = *c*_0_ + *c*_1_ e^(−*x*/*t*)^where, *c*_0_ and *c*_1_ are fitting constants with values 6.127(5) Å and 0.004(5) Å, respectively and decaying constant (*t*) is 0.08(3). The unit cell volume varies with the concentrations of Bi^3+^ ions according to the expression:*V* = *V*_0_ + *V*_1_ e^(−*x*/*t*)^where, *V*_0_ and *V*_1_ are fitting parameters with values *V*_0_ = 82.054(4) Å^3^, *V*_1_ = 0.178(6) Å^3^, respectively and *t* is decaying constant with *t* = 0.031(4). The average values of crystallite size and lattice strain for the Bi^3+^ doped La^3+^/Er^3+^/Yb^3+^ nano-phosphor samples have been estimated using Williamson–Hall (W–H) method. The W–H plot method is expressed by the following equation:*β*_*hkl*_ cos *θ*_*hkl*_ = 0.89*λ*/*d* + 4*ε* sin *θ*_*hkl*_where, *β*_*hkl*_ is full width at half maxima (FWHM) for (*hkl*) peak, *d* is the average value of crystallite size, *λ* is the wavelength of radiation used in XRD measurement (*λ* = 1.5406 Å), *ε* is average value of lattice strain and *θ*_*hkl*_ is Bragg's angle for (*hkl*) reflection.^34^ Ball and Stick models of the unit cells for space groups (a) *P*3̄*m*1 and (b) *R*3̄*m* have been drawn to see the arrangement of lanthanum and oxygen atoms (see Fig. 4). A representative W–H plot for the Er^3+^/Yb^3+^ co-doped La_2_O_3_ phosphor sample is shown in Fig. 5(a). The average values of crystallite size have been calculated and are found to be 55.9, 65.1, 66.3 and 88.9 nm for the 0, 5, 10 and 15 mol% concentrations of Bi^3+^ ions in the nano-phosphor samples, respectively for La_2_O_3_ phase with space group *P*3̄*m*1. The crystallite size increases with increasing the concentrations of Bi^3+^ ions. The average values of the lattice strain increases from 5.3 × 10^−4^ to 2.2 × 10^−3^ for the concentrations of Bi^3+^ ions from 0 to 15 mol%, respectively. The variation in lattice strain for the La_2_O_3_ phase as a function of different concentrations of Bi^3+^ ions is shown in Fig. 5(b).

**Table 1:** The structural lattice parameters obtained after Rietveld analysis of the Er^3+^/Yb^3+^/*x*Bi^3+^ tri-doped La_2_O_3_ nano-phosphors (*i.e. x* = 0, 5, 10 and 15 mol%) using space groups *P*3̄*m*1 and (*P*3̄*m*1 + *R*3̄*m*)

Bi^3+^ (mol%)	*P*3̄*m*1			*R*3̄*m*			*χ* ^2^
	*a* = *b*(Å)	*c*(Å)	*V*(Å)	*a* = *b*(Å)	*c*(Å)	*V*(Å)	
0	3.9356(1)	6.1306(2)	82.233(2)				1.79
5	3.9326(1)	6.1291(2)	82.088(4)				1.81
10	3.9324(1)	6.1280(3)	82.065(6)	3.9548(2)	9.9279(9)	134.48(2)	1.86
15	3.9322(1)	6.1277(3)	82.053(6)	3.9531(2)	9.9193(6)	134.24(1)	1.73

The detailed information of the estimated values for the crystallite size and the lattice strain are tabulated in Table 2.

**Table 2:** Crystallite size and lattice strain obtained from Williamson–Hall method of the Er^3+^/Yb^3+^/*x*Bi^3+^ tri-doped La_2_O_3_ nano-phosphors (*i.e. x* = 0, 5, 10 and 15 mol%)

Concentration of Bi^3+^ (*x* mol%)	Phase 1 (*P*3̄*m*1)		Phase 2 (*R*3̄*m*)	
	*d* (nm)	*ε*	*d* (nm)	*ε*
0	55.9	5.3 × 10^−4^		
5	65.1	1.5 × 10^−3^		
10	66.3	1.6 × 10^−3^	69.3	6.9 × 10^−4^
15	88.9	2.2 × 10^−3^	73.7	8.4 × 10^−3^

#### 3.1.2 SEM and EDS measurements

**Fig. 6:**
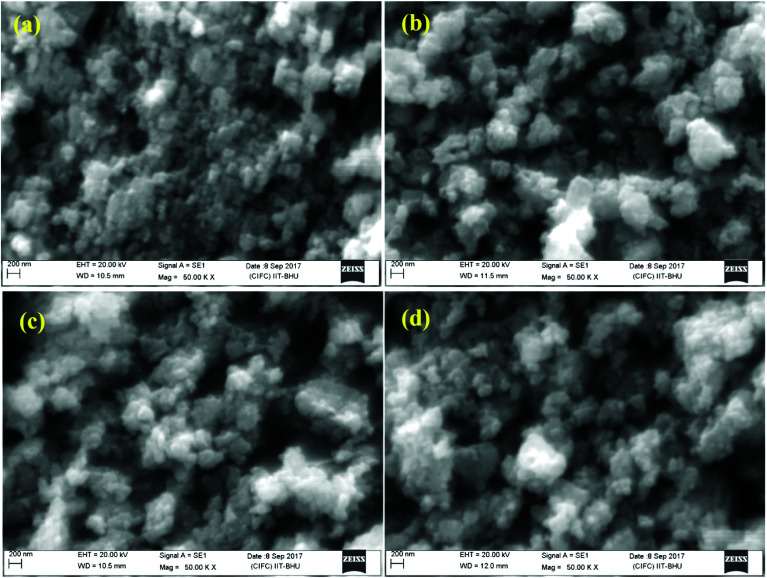
SEM micrographs of the Er^3+^/Yb^3+^/*x*Bi^3+^ tri-doped nano-phosphor samples with *x* = (a) 0 mol%, (b) 5 mol%, (c) 10 mol% and (d) 15 mol% concentrations of Bi^3+^ ions.

Fig. 6 shows scanning electron microscopy (SEM) micrographs for different concentrations of Bi^3+^ ions (*i.e. x* = 0, 5, 10, and 15 mol%) in the Er^3+^/Yb^3+^/*x*Bi^3+^ tri-doped La_2_O_3_ nano-phosphor samples. The average particles size for all the samples were estimated considering large number of particles using ImageJ software. Its average values are found to be 101, 113, 118 and 141 nm for *x* = 0, 5, 10 and 15 mol% concentrations of Bi^3+^ ions in the Er^3+^/Yb^3+^/*x*Bi^3+^ tri-doped La_2_O_3_ phosphors, respectively. The particles of the phosphor are almost spherical in shape and agglomerated to each other in different orientations. It is also clear from Fig. 6(a–d) that the particles size of the phosphor increases on addition of Bi^3+^ ions.^15,27^

**Fig. 7:**
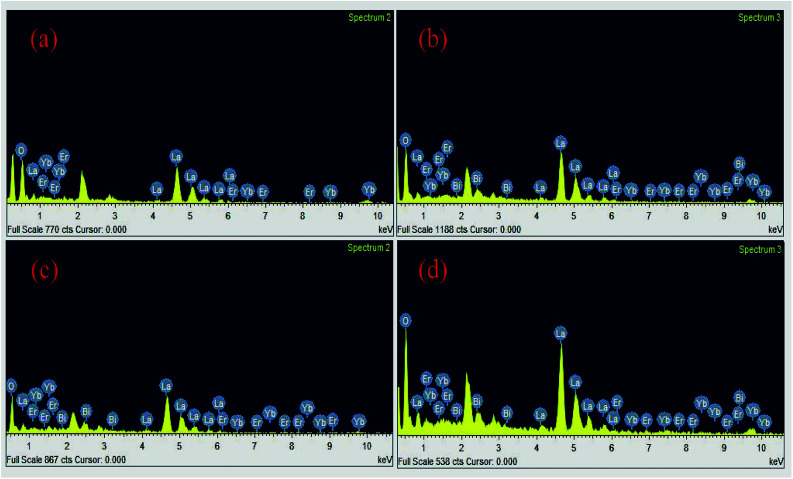
The EDS spectra of the Er^3+^/Yb^3+^/*x*Bi^3+^ tri-doped samples with *x* = (a) 0 mol% (b) 5 mol% (c) 10 mol% and (d) 15 mol% concentrations of Bi^3+^ ions.

Fig. 7 shows the energy dispersive spectroscopic (EDS) patterns of the Er^3+^/Yb^3+^/*x*Bi^3+^ tri-doped La_2_O_3_ nano-phosphor samples for different concentrations of Bi^3+^ ions (*i.e.* 0, 5, 10 and 15 mol%). Since, the EDS technique used in conjunction with SEM for elemental analysis is not much suitable for lighter elements like oxygen; hence, we report only their qualitative analysis rather than quantitative. The EDS spectra reveal the presence of La, Er, Yb, Bi and O elements in the tri-doped nano-phosphor samples.^15^ As is clear from Fig. 7(a) that the Er^3+^/Yb^3+^ co-doped La_2_O_3_ nano-phosphor contains only La, Yb, Er and O elements in the sample. When the Bi^3+^ ion is added in the co-doped phosphor it contains an additional Bi element along with La, Er, Yb and O elements (see Fig. 7(b–d)).

### 3.2 Optical characterization

#### 3.2.1 FTIR measurements

**Fig. 8:**
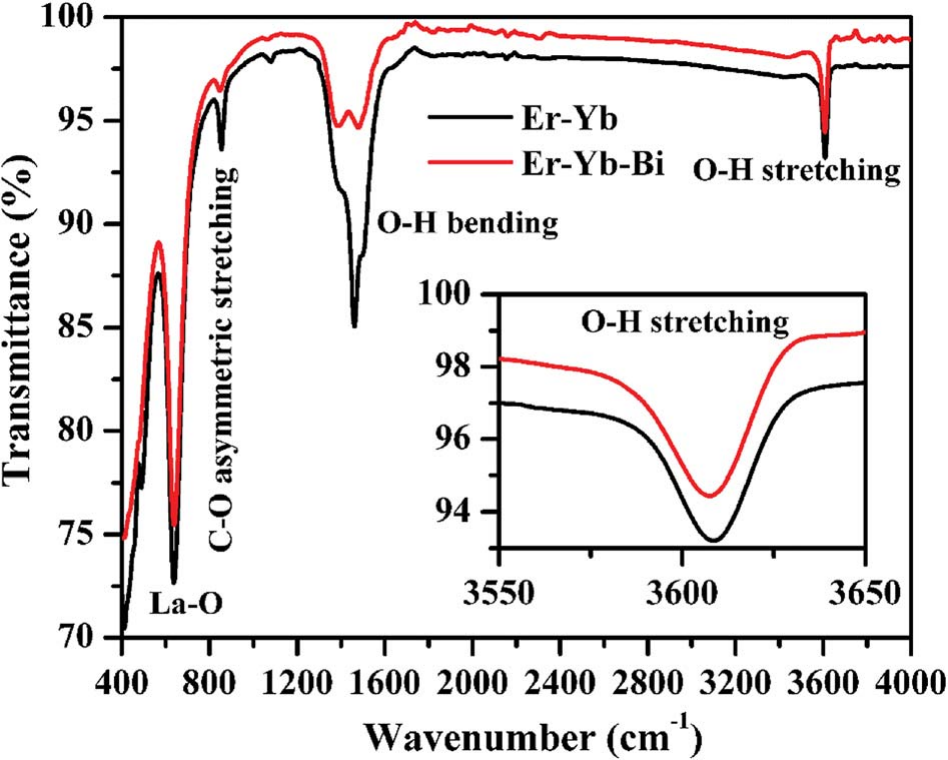
FTIR spectra of the Er^3+^/Yb^3+^/*x*Bi^3+^ tri-doped La_2_O_3_ nano-phosphors for different concentrations of Bi^3+^ ions (*i.e. x* = 0 and 5 mol%). The inset figure shows the zoomed part (3550–3650 cm^−1^) of stretching vibration of the O–H molecule.

The FTIR spectra of the Er^3+^/Yb^3+^/*x*Bi^3+^ tri-doped La_2_O_3_ phosphor samples were recorded in the range of 400–4000 cm^−1^ for *x* = 0 and 5 mol% concentrations of Bi^3+^ ions and the spectra thus obtained are shown in Fig. 8. The spectra show peaks centred at 411, 490 and 638 cm^−1^ in the 400–650 cm^−1^ region and they are assigned to be stretching vibrations of La–O molecule, respectively.^8,15^ The spectra also contain a peak at 855 cm^−1^ due to asymmetric stretching vibration of the C–O molecule. Since the samples have been synthesized through solution combustion method, the spectra contain two peaks due to hydroxyl group at 1462 and 3615 cm^−1^ corresponding to the bending and stretching vibrations of H_2_O content, respectively. The presence of O–H group has been also verified from the XRD patterns in the form of La(OH)_3_. The La(OH)_3_ phase is present in a very small amount and can decrease the emission intensity.^8^ The inset figure shows the zoomed part of the stretching vibration of O–H molecule for the lattice vibration of 3615 cm^−1^. It is also clear from the figure that the intensity of O–H bands decreases considerably in presence of Bi^3+^ ions. This supports the fact that on increasing the doping concentrations of Bi^3+^ ions the emission intensity arising from the thermally coupled levels of Er^3+^ ion could be increased.

#### 3.2.2 Absorption measurements and optical band gap calculation

**Fig. 9:**
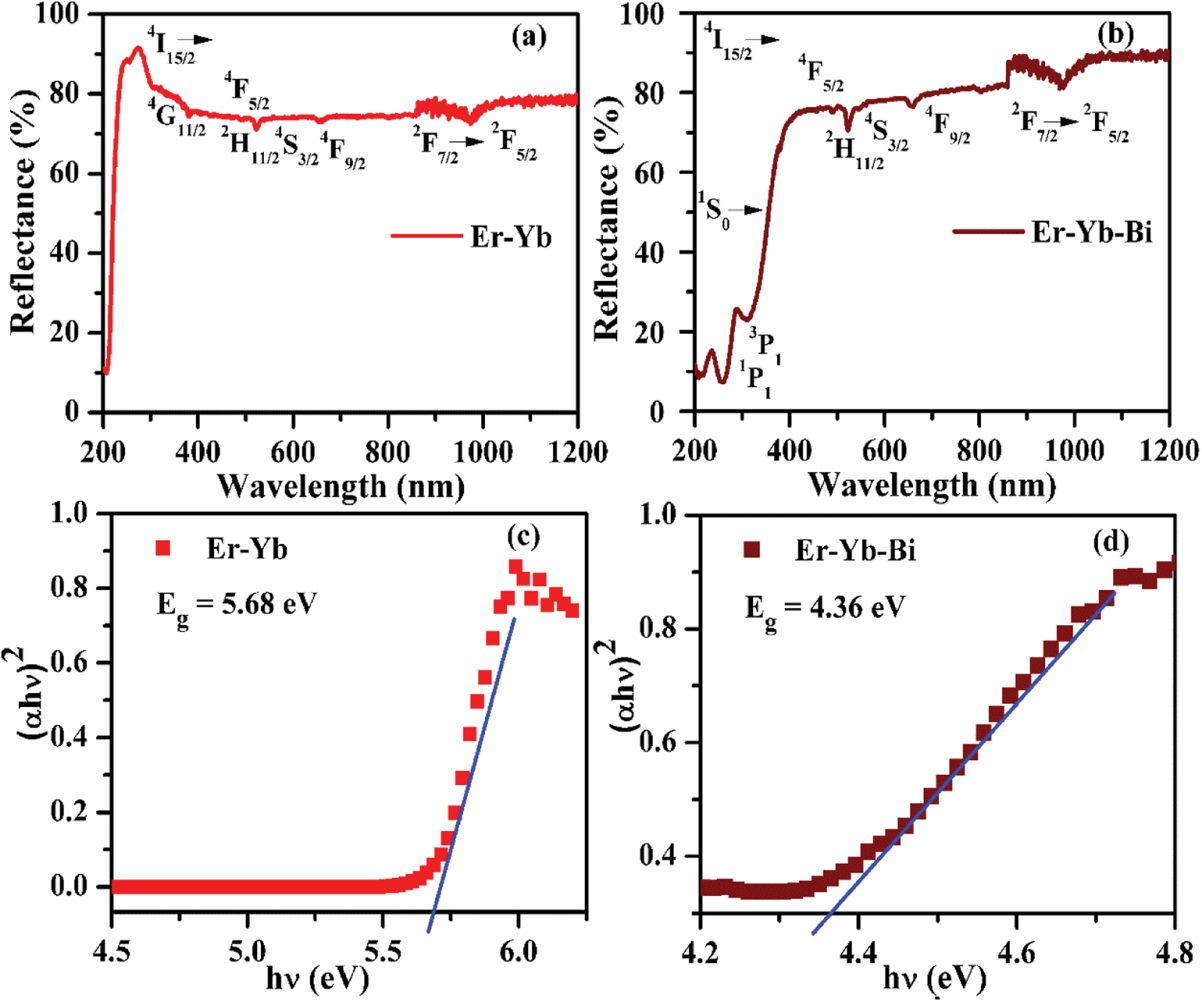
The absorption spectra (a and b) and the optical band gaps (c and d) of the Er^3+^/Yb^3+^/*x*Bi^3+^ tri-doped La_2_O_3_ nano-phosphors (*i.e. x* = 0 and 5 mol%).

The UV-Vis-NIR absorption spectra of the Er^3+^/Yb^3+^/*x*Bi^3+^ tri-doped La_2_O_3_ phosphor samples monitored in the 200–1200 nm region for *x* = 0 and 5 mol% concentrations of Bi^3+^ ions are shown in Fig. 9(a and b). The spectra contain various absorption bands due to Er^3+^, Yb^3+^ and Bi^3+^ ions. Fig. 9(a) shows large number of peaks at 381, 490, 523, 548 and 671 nm and they are attributed to ^2^I_15/2_ → ^4^G_11/2_, ^4^F_5/2_, ^2^H_11/2_, ^4^S_3/2_ and ^4^F_9/2_ transitions of Er^3+^ ion, respectively.^12–18^ The figure also shows a broad band centered at 976 nm due to ^2^F_7/2_ → ^2^F_5/2_ transition of Yb^3+^ ion.^15^ When the Bi^3+^ ion is added in the phosphor the spectrum contains additional bands in the 220–340 nm region due to Bi^3+^ ion (see Fig. 9(b)). The bands seen at 253 and 310 nm are due to ^1^S_0_ → ^1^P_1_ and ^1^S_0_ → ^3^P_1_ transitions of the Bi^3+^ ion, respectively.^15^ It is also clear from Fig. 9(a and b) that there is no absorption band due to the host. The broad band observed in the NIR region proves the larger absorption cross section of Yb^3+^ ion for NIR light (*i.e.* 976 nm). This could be favorable for larger emission intensity.

The optical band gap has been calculated for the La_2_O_3_ phosphors in both the cases using Wood and Tauc formula:^35^
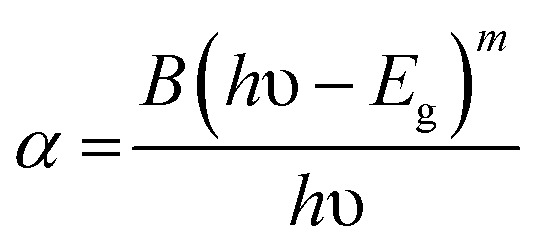
where *E*_g_, *hυ* and *B* are the band gap energy, the incident photon energy and band tailoring constant, respectively. The value of ‘*m*’ has been used as (1/2) for direct band gap allowed transition.^10,15^ The (*αhυ*)^2^*versus hυ* curves have been plotted in both the cases and the obtained curves are shown in Fig. 9(c and d). The optical band gap is calculated as 5.68 eV in the pure La_2_O_3_ phosphor (see Fig. 9(c)) and matches to the reported value 5.80 eV.^15^ The value of optical band gap is reduced considerably in presence of Bi^3+^ ions and it is 4.36 eV (see Fig. 9(d)). It has been also reported that the optical band gap of the material was decreased in presence of Bi^3+^ ion.^36,37^ The Bi^3+^ ion creates some energy levels as a continuous band with the 6s^2^ valance electrons. We have also observed similar effect of Bi^3+^ doping on the optical band gap of the phosphor materials.^15,38^ It is also clear from the Fig. 9(c and d) that the optical band gap greatly depends on the concentrations of Bi^3+^ ions. The decrease in optical band gap may promote large number of the excited ions in the upper energy levels. As a result, one can achieve large emission intensity in presence of Bi^3+^ ions.

#### 3.2.3 Upconversion measurements

**Fig. 10:**
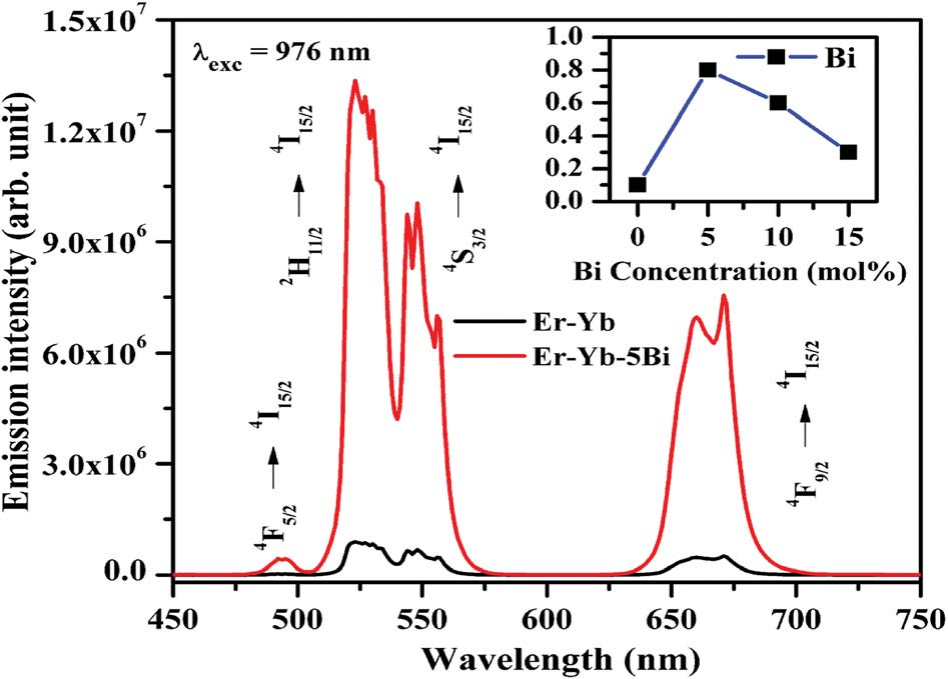
The upconverted emission spectra of the Er^3+^/Yb^3+^/*x*Bi^3+^ tri-doped La_2_O_3_ nano-phosphors (where *x* = 0 and 5 mol%) on excitation with 976 nm. The inset figure shows the effect of Bi^3+^ ion concentration on the emission intensity of the Er^3+^/Yb^3+^ co-doped La_2_O_3_ nano-phosphor samples.

The upconverted emission spectra of the Er^3+^/Yb^3+^/*x*Bi^3+^ tri-doped La_2_O_3_ nano-phosphor samples (where *x* = 0, 5, 10, and 15 mol%) have been recorded in the spectral range of 450–750 nm using 976 nm excitation source and the spectra thus obtained are shown in Fig. 10. The phosphor sample gives a weak blue (at 492 nm), strong green (at 523 and 548 nm) and red (at 660 and 671 nm) upconversion emissions due to ^4^F_5/2_ → ^4^I_15/2_, ^2^H_11/2_ → ^4^I_15/2_, ^4^S_3/2_ → ^4^I_15/2_, and ^4^F_9/2_ → ^4^I_15/2_ transitions of Er^3+^ ion, respectively.^12–18^ The blue emission arises due to absorption of three 976 nm photons whereas the green and red emissions have been observed due to absorption of two 976 nm photons, respectively.^15^ The emission intensity of the phosphors has been measured for different concentrations of Bi^3+^ ions while the concentrations of Er^3+^ and Yb^3+^ ions are fixed at 0.7 and 3.0 mol%, respectively. It has been observed that the emission intensity of Er^3+^ ion initially increases on increasing the concentrations of Bi^3+^ ions upto 5 mol%. After this, the emission intensity of the sample decreases due to concentration quenching. This is due to the fact that at higher concentrations, the distance between the ions becomes smaller compared to their critical distance and the excitation energy migrates to the quenching sites.^10,15^ The effect of Bi^3+^ ion concentration on the emission intensity of the Er^3+^/Yb^3+^ co-doped La_2_O_3_ phosphors is shown as inset in Fig. 10. The figure shows that the emission intensity is found to be optimum for 5 mol% concentrations of Bi^3+^ ions. The figure also shows that the emission intensity of the co-doped phosphor is enhanced upto 15 times in presence of Bi^3+^ ions.

There may be several reasons for an increase in the emission intensity of Er^3+^ ion in the phosphor sample. The initial reason is that when the Bi^3+^ ion is co-doped in the phosphor; the crystallite size of the phosphor increases from 55.9 to 65.1 nm. This improves the crystallinity of the phosphor sample. The effect of Bi^3+^ ion concentration on the crystallinity of the phosphor has been studied by Choudhary *et al.* and they have found an enhancement in the emission intensity of the dopant ions due to improvement in crystallinity.^27^ It has been also mentioned in the XRD section that the phase of the phosphor remains the same on co-doping of 5 mol% concentrations of Bi^3+^ ions and we have also found optimum emission intensity in this case.^15^ When the concentrations of Bi^3+^ ion is increased further from 10 to 15 mol% a small amount of an additional rhombohedral phase of BiLa_2_O_4.5+*δ*_ was observed in the phosphor sample. The presence of an additional phase creates large strain in the phosphor and reduces the emission intensity significantly for higher concentrations of Bi^3+^ ions. The other possibility is due to an increase in the particles size of the phosphor *via* Bi^3+^ doping. As has been observed in the SEM micrographs that the particles size of the phosphor increases as the concentration of Bi^3+^ ions is increased.^15,27^ The FTIR analysis also shows a reduction in the intensity of O–H bands significantly in presence of Bi^3+^ ion. It has been also reported that the presence of Bi^3+^ ion increases the lifetime of the emitting levels, which supports the larger photoluminescence intensity for the phosphor samples.^15,38^ The optical band gap analysis also shows a reduction in the optical band gap from 5.68 to 4.36 eV in presence of Bi^3+^ ion. This leads to promote large number of excited ions in the upper energy levels, thereby emitting large photoluminescence intensity. Thus, in our case we could observe an enhancement in the emission intensity upto 15 times in presence of Bi^3+^ ion due to these combined effects.

**Fig. 11:**
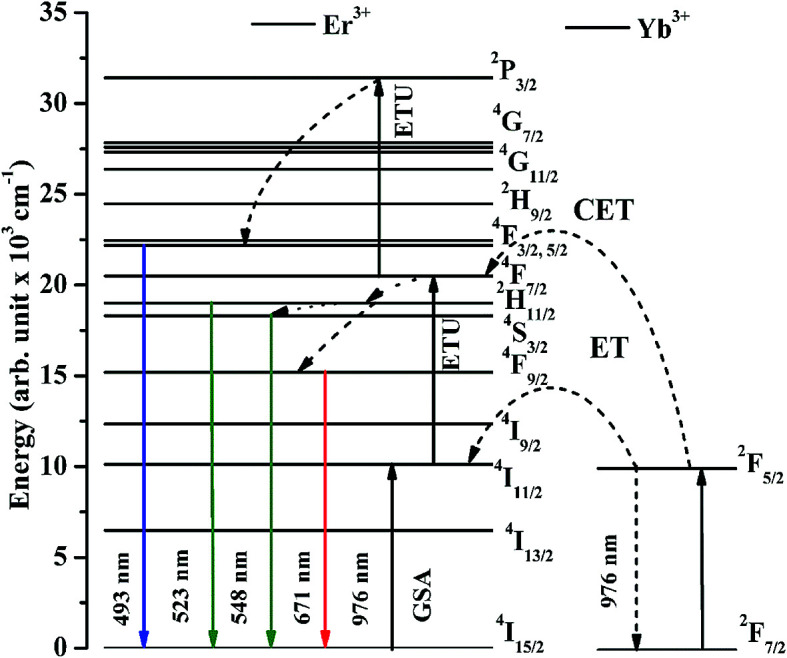
Energy level diagrams of the Er^3+^ and Yb^3+^ ions and the excitation and emission processes in them.

The excitation and emission processes involved in Er^3+^ and Yb^3+^ ions and energy transfer between them are shown in Fig. 11. The figure shows that the ground state absorption (GSA), energy transfer upconversion (ETU) and cooperative energy transfer (CET) processes are observed effectively. As the Er^3+^, Yb^3+^ co-doped La_2_O_3_ sample is excited by 976 nm the Yb^3+^ ions are promoted from their ground state (^2^F_7/2_) to the excited state (^2^F_5/2_) due to large absorption cross section for this wavelength. The excited Yb^3+^ ions transfer their excitation energy in the ground state (^4^I_15/2_) of Er^3+^ ions on relaxation. Due to this, the Er^3+^ ions are excited to ^4^I_11/2_ state. The Er^3+^ ions are also excited directly to ^4^I_11/2_ state weakly through GSA due to absorption of 976 nm photons. These ions are further excited to ^4^F_7/2_ state due to absorption of another photon through ETU process. On the other hand, the two Yb^3+^ ions transfer their energy simultaneously to Er^3+^ ions in the ground state (^4^I_15/2_) due to CET, which promote the Er^3+^ ions to ^4^F_7/2_ state also. This facilitates large number of the excited ions in ^4^F_7/2_ state and some of the ions are further promoted to ^2^P_3/2_ state through ETU process. Finally, the ^4^F_5/2,_^2^H_11/2_, ^4^S_3/2_ and ^4^F_9/2_ states are populated, which give radiative transitions at 493, 523, 548 and 671 nm wavelengths, respectively.^12–18,20^

#### 3.2.4 Induced optical heating measurements

**Fig. 12:**
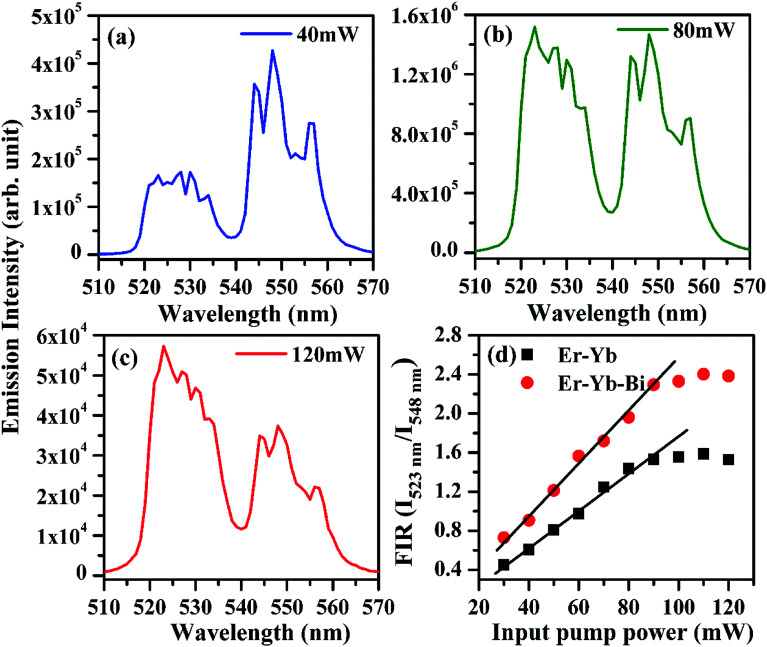
Effect of incident pump powers on the upconverted emission intensity of the thermally coupled levels (^2^H_11/2_ and ^4^S_3/2_) of the Er^3+^ ion in the Er^3+^/Yb^3+^/5Bi^3+^ tri-doped La_2_O_3_ nano-phosphor (a–c) and (d) FIR of the Er^3+^/Yb^3+^/*x*Bi^3+^ tri-doped La_2_O_3_ nano-phosphors (*i.e. x* = 0 and 5 mol%) on excitation with 976 nm.

The induced optical heating in the Er^3+^/Yb^3+^ co-doped phosphors have been widely studied by different groups of researchers in different host materials.^4–6,19^ They have measured the emission intensity of the thermally coupled levels (^2^H_11/2_ and ^4^S_3/2_) of the Er^3+^ ion at various pump powers and observed a change in emission intensities of the two bands arising from them to a common lower level. We have monitored the emission intensities for the two emission bands of Er^3+^ ion in the Er^3+^/Yb^3+^/5Bi^3+^ tri-doped La_2_O_3_ phosphor at various pump powers of 976 nm excitation and they are shown in Fig. 12. As is clear from Fig. 12 that the intensity of band lying at lower wavelength (*λ*) side is smaller than that of at higher (*λ*) side. As the pump power is increased, the excited ions are promoted from the ^4^S_3/2_ level to the ^2^H_11/2_ level due to heating effect. Therefore, the intensity of band in lower (*λ*) side increases and very soon its intensity becomes larger than the other bands. The fluorescence intensity ratio (FIR) of the two bands increases with pump power. The heating arises due to an increase in pump power. Addition of surface modifiers can also affect the FIR due to increase in emission intensities of the thermally coupled peaks.^24–27^

It is clear from the figure that the emission intensity of the 523 nm band due to ^2^H_11/2_ → ^4^I_15/2_ transition is smaller than that of 548 nm band due to ^4^S_3/2_ → ^4^I_15/2_ transition, respectively at 40 mW pump power. It shows that initially, the population of the ions in the excited ^2^H_11/2_ level is smaller than that of ^4^S_3/2_ level at lower pump power. It also shows that the phosphor sample emits very intense green photoluminescence even at very low pump power (see Fig. 12(a)). As the pump power is increased the excited ions are promoted from ^4^S_3/2_ level to ^2^H_11/2_ level. As a result, the emission intensity of the peak arising from ^2^H_11/2_ level increases considerably relative to the intensity of the peak arising from ^4^S_3/2_ level and reaches to almost in equal values at 80 mW pump power (see Fig. 12(b)). When the pump power was increased further from 80 to 120 mW the overall intensity of both the emission bands is reduced due to the sample heating. However, the emission intensity of the peak arising from the ^2^H_11/2_ thermally coupled level is increased while the emission intensity of the peak arising from the ^4^S_3/2_ thermally coupled level is found to decrease. Fig. 12(c) shows that the emission intensity of the peak arising from ^2^H_11/2_ level (523 nm) is larger than that of ^4^S_3/2_ level (548 nm) at 120 mW pump power. The variation in emission intensities has been used to calculate the fluorescence intensity ratio (*i.e.* FIR = *I*_523 nm_/*I*_548 nm_) in absence and presence of 5 mol% Bi^3+^ ions (see Fig. 12(d)). The FIR *versus* input pump power plot shows almost linear behavior upto 90 mW in both the cases. However, the plots show a deviation from linear behavior for higher pump powers *i.e.* greater than 90 mW. This occurs due to optical heating in the sample at higher pump powers. The FIR of the phosphor is found to be sustaining higher values of pump power in presence of Bi^3+^ ions. Thus, the tri-doped phosphor may provide a platform for the study of optical heating based applications.

#### 3.2.5 Temperature sensing measurements of the nano-phosphor

**Fig. 13:**
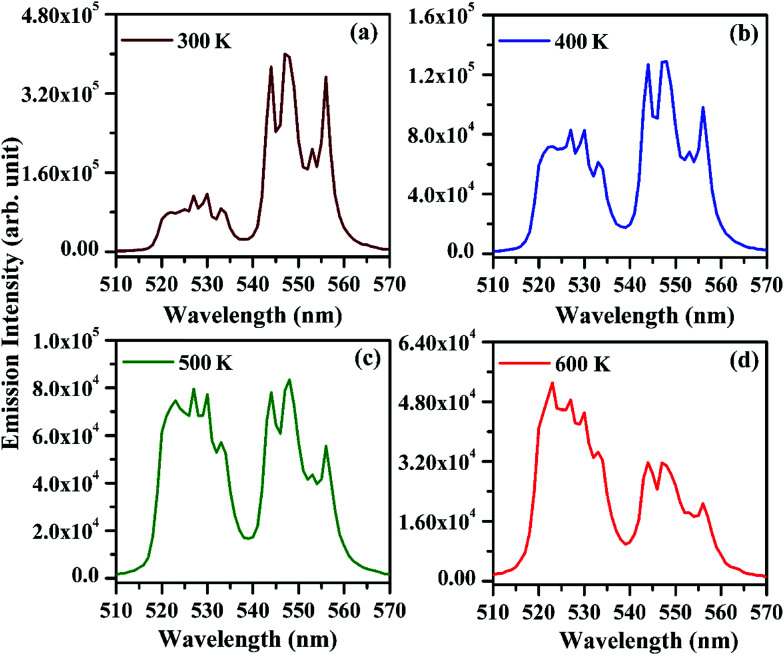
Effect of temperature on emission intensities of the thermally coupled levels (*i.e.* 523 and 548 nm emission bands) of the Er^3+^/Yb^3+^/5Bi^3+^ tri-doped La_2_O_3_ nano-phosphor upon 976 nm excitation excited at 40 mW.

As has been observed earlier, the emission intensity of the bands arising from the thermally coupled levels (^2^H_11/2_ and ^4^S_3/2_) of the Er^3+^ ion varies significantly on increasing the pump power. The increase in pump power heats the sample due to non-radiative relaxations and thereby affects the intensity of the bands arising from the two levels. We have measured the emission intensity of the peaks arising from the thermally coupled levels by heating the Er^3+^/Yb^3+^/*x*Bi^3+^ tri-doped La_2_O_3_ phosphor samples (*i.e. x* = 0 and 5 mol%) externally in the range of 300–625 K on excitation with 976 nm at 40 mW. The effect of temperature on the emission intensities of the thermally coupled levels of Er^3+^ ion in the Er^3+^/Yb^3+^/5Bi^3+^ tri-doped La_2_O_3_ phosphor on excitation with 976 nm at 40 mW is shown in Fig. 13. The emission intensity of the bands arising from the thermally coupled levels (*i.e.*^2^H_11/2_ and ^4^S_3/2_) of the Er^3+^ ion at 523 and 548 nm wavelengths differs from each other at room temperature.^4–6,24–27^ Initially, the emission intensity of 523 nm band is less than that of 548 nm band at 300 K (see Fig. 13(a)). Fig. 13(b) shows significant increase in the emission intensity of 523 nm band at 400 K. However, at 500 K the emission intensities of the two bands are found almost the same (see Fig. 13(c)). As temperature of the phosphor sample further increases, the overall emission intensity of the 523 and 548 nm emission bands decreases due to thermal quenching. Actually, the increase in temperature of the sample leads to increase the lattice vibrations of the ions and thereby non-radiative transitions, which decreases the emission intensity. The relative intensity of the two emission bands changes accordingly and the emission intensity of the 523 nm band is larger compared to that of 548 nm band at 600 K (see Fig. 13(d)). The variation in emission intensity is due to transfer of the excited ions from the lower to the upper thermally coupled levels *via* the lattice vibrations. This increases the population of ions in the upper component of the thermally coupled level and enhances the emission intensity of 523 nm band. The similar observation has been made by Dey *et al.* in the Er^3+^/Yb^3+^ co-doped phosphor.^11^ The figures clearly show that there is a variation in emission intensities of the two bands arising from the thermally coupled levels of the Er^3+^ ion.

**Fig. 14:**
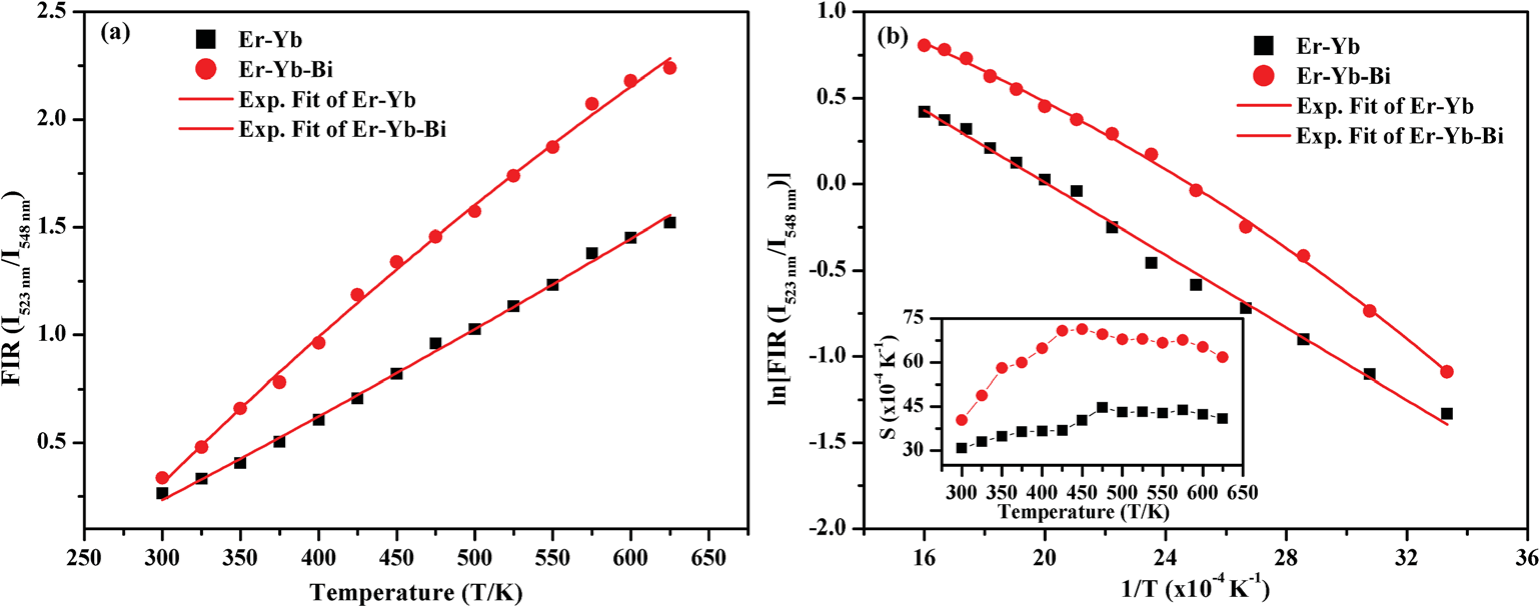
(a) Plots of FIR(*I*_523 nm_/*I*_548 nm_) *versus* temperature, (b) ln(FIR(*I*_523 nm_/*I*_548 nm_)) *versus T*^−1^ and inset in (b) shows sensitivity *versus* temperature for the Er^3+^/Yb^3+^/*x*Bi^3+^ tri-doped La_2_O_3_ nano-phosphors on excitation with 976 nm laser at 40 mW (*i.e. x* = 0 and 5 mol%).

The FIR values have been evaluated using the values of emission intensities of 523 and 548 nm bands as a function of temperature and the plot thus obtained between FIR and the temperature is shown in Fig. 14(a). It has been reported by Pérez-Rodríguez *et al.*^39^ that the non-radiative relaxations of the ions arising between the two levels conserve them thermally coupled. The theoretically calculated energy gap value is ∼800 cm^−1^ and population of the ions in the thermally coupled levels seems to follow Boltzmann population distribution law.^11^ The fluorescence intensity ratio (FIR) of the two thermally coupled ^2^H_11/2_ and ^4^S_3/2_ levels of the Er^3+^ ion is related by the following relation:

i FIR = *I*_523 nm_/*I*_548 nm_ = *R* exp(−Δ*E*/*kT*)

where *I*_523 nm_ and *I*_548 nm_ are the emission intensities of the thermally coupled ^2^H_11/2_ and ^4^S_3/2_ levels, respectively. The *R* and Δ*E* are the proportionality constant and energy gap between the two sublevels, respectively. The *k* and *T* are Boltzmann's constant and absolute temperature of the phosphor sample, respectively.^4–6^

It is also clear from the Fig. 14(a) that the FIR of the thermally coupled ^2^H_11/2_ and ^4^S_3/2_ levels increases non-linearly for the Er^3+^/Yb^3+^/*x*Bi^3+^ tri-doped La_2_O_3_ phosphor samples (*i.e. x* = 0 and 5 mol%), respectively as the temperature of the phosphor is increased from 300 to 625 K. The presence of Bi^3+^ ion in the co-doped phosphor gives the larger FIR with the increase in temperature. We have also plotted a graph between the logarithmic of FIR and the inverse of temperature (*i.e. T*^−1^) and it is shown in Fig. 14(b). The ln(FIR) *versus T*^−1^ plots were fitted in eqn (i), which give the slope values as 1051 and 1077 for the co-doped and tri-doped phosphors, respectively. We have calculated the energy separation between the two thermally coupled levels using these values and they are found to be 730 and 749 cm^−1^ for the co-doped and tri-doped phosphor samples, respectively. The calculated values thus obtained in our case are very close to the earlier reported value (*i.e.* 738.54 cm^−1^).^26^

The temperature sensitivity of the phosphor sample is an important term for highly sensitive optical temperature sensors. The sensitivity (*S*) is obtained through a variation of FIR with the increase in temperature of the phosphor sample and it can be calculated using the following expression:^24–27^

ii *S* = d(FIR)/d*T* = FIR(Δ*E*/*kT*^2^)

where the terms have their usual meanings.

The plots between the sensitivity and the temperature were obtained using relation (ii) for the measured range of temperatures *i.e.* 300–625 K in absence and presence of Bi^3+^ ions and it is shown as inset in Fig. 14(b). The inset of Fig. 14(b) shows that the sensitivity varies as the temperature of the phosphor is increased. The relative sensitivity of the Er^3+^/Yb^3+^ co-doped phosphor sample varies from 31 × 10^−4^ K^−1^ to 41 × 10^−4^ K^−1^ on increasing the temperature from 300 to 625 K, respectively and the maximum relative sensitivity is found to be 45 × 10^−4^ K^−1^ at 475 K. Similarly, the relative sensitivity of the Er^3+^/Yb^3+^/Bi^3+^ tri-doped phosphor sample initially increases with the increase in temperature and then it is found to decrease after reaching to its maximum value as 71 × 10^−4^ K^−1^ at 450 K.^24^ The presence of Bi^3+^ ion also improves the temperature sensitivity of the Er^3+^/Yb^3+^ co-doped La_2_O_3_ phosphor significantly, which is due to improvement in the local crystal structure around the ions. The sensitivity observed in our case has been compared with the earlier reported values in different host materials and they are given in Table 3.^24,40–44^ The table shows that the value of sensitivity observed in our case is found to be larger than the earlier reported values in different host materials. Therefore, the Er^3+^/Yb^3+^/Bi^3+^ tri-doped La_2_O_3_ nano-phosphor may be used in the temperature sensing applications.

**Table 3:** The temperature sensitivity observed in different Er^3+^/Yb^3+^ activated host materials

Materials	Temperature range	Sensitivity (*S*)	Ref.
Er^3+^, Yb^3+^ co-doped Gd_2_O_3_	300–900 K	39 × 10^−4^ K^−1^ at 300 K	40
Er^3+^, Yb^3+^ co-doped Y_2_SiO_5_	300–600 K	56 × 10^−4^ K^−1^ at 400 K	41
Er^3+^, Yb^3+^ co-doped NaYF_4_	273–333 K	42 × 10^−4^ K^−1^ at 328 K	42
Er^3+^, Yb^3+^, Li^+^ tri-doped Na_2_Zn_2_PO_4_	300–603 K	65 × 10^−4^ K^−1^ at 603 K	43
Er^3+^, Yb^3+^ co-doped La_2_O_3_	300–625 K	45 × 10^−4^ K^−1^ at 475 K	Present work
Er^3+^, Yb^3+^, Bi^3+^ tri-doped La_2_O_3_	300–625 K	71 × 10^−4^ K^−1^ at 450 K	Present work
Er^3+^, Yb^3+^, Li^+^ tri-doped Y_2_Ti_2_O_7_	298–673 K	67 × 10^−4^ K^−1^ at 363 K	24
Er^3+^, Yb^3+^ co-doped NaLnTiO_4_	300–510 K	45 × 10^−4^ K^−1^ at 573 K	44

## 4. Conclusions

The Er^3+^/Yb^3+^/Bi^3+^ tri-doped La_2_O_3_ nano-phosphor has been synthesized through solution combustion method. The structural measurements of the phosphor show an improvement in the local crystal structure in presence of Bi^3+^ ions. The lattice parameters and the particles size of the phosphor increase on increasing the Bi^3+^ concentrations. The Er^3+^/Yb^3+^ co-doped phosphor emits a weak blue, strong green and red upconverted emission bands upon 976 nm excitation. The emission intensity of the co-doped phosphor sample is enhanced upto 15 times in presence of Bi^3+^ ion due to combined effect of an increase in crystallinity, particles size and reduction in optical quenching centers. The fluorescence intensity ratio (FIR) of the thermally coupled levels shows an efficient induced optical heating in the phosphor. The FIR of the 523 and 548 nm emission bands also varies with the increase in temperature of the phosphor. The relative temperature sensing sensitivity is calculated to be 71 × 10^−4^ K^−1^ at 450 K. Thus, the Er^3+^/Yb^3+^/Bi^3+^ tri-doped La_2_O_3_ nano-phosphor may be suitable candidate for the photonic devices, as an optical heater and temperature sensor.

## Conflicts of interest

The authors declare that there is no conflict of interest in the manuscript.

## Supplementary Material
